# Photodeposition of Silver on Zinc/Calcium Ferrite Nanoparticles: A Contribution to Efficient Effluent Remediation and Catalyst Reutilization

**DOI:** 10.3390/nano11040831

**Published:** 2021-03-24

**Authors:** Ricardo J. C. Fernandes, Carlos A. B. Magalhães, Ana Rita O. Rodrigues, Bernardo G. Almeida, Ana Pires, André Miguel Pereira, João Pedro Araujo, Elisabete M. S. Castanheira, Paulo J. G. Coutinho

**Affiliations:** 1 Physics Center of Minho and Porto Universities (CF-UM-UP), University of Minho, Campus de Gualtar, 4710-057 Braga, Portugal; rica45fernandes@gmail.com (R.J.C.F.); pg30870@alunos.uminho.pt (C.A.B.M.); ritarodrigues@fisica.uminho.pt (A.R.O.R.); bernardo@fisica.uminho.pt (B.G.A.); ecoutinho@fisica.uminho.pt (E.M.S.C.); 2 IFIMUP-Instituto de Física dos Materiais, Universidade do Porto, R. Campo Alegre, 4169-007 Porto, Portugal; ana.pires@fc.up.pt (A.P.); ampereira@fc.up.pt (A.M.P.); jearaujo@fc.up.pt (J.P.A.)

**Keywords:** zinc/calcium ferrite, silver photodeposition, photodegradation, azo dyes

## Abstract

The efficient photodegradation of textile dyes is still a challenge, especially considering resistant azo dyes. In this work, zinc/calcium mixed ferrite nanoparticles prepared by the sol–gel method were coupled with silver by a photodeposition method to enhance the photocatalytic potency. The obtained zinc/calcium ferrites are mainly cubic-shaped nanoparticles sized 15 ± 2 nm determined from TEM and XRD and an optical bandgap of 1.6 eV. Magnetic measurements indicate a superparamagnetic behavior with saturation magnetizations of 44.22 emu/g and 27.97 emu/g, respectively, for Zn/Ca ferrite and Zn/Ca ferrite with photodeposited silver. The zinc/calcium ferrite nanoparticles with photodeposited silver showed efficient photodegradation of the textile azo dyes C.I. Reactive Blue 250 and C.I. Reactive Yellow 145. Subsequent cycles of the use of the photocatalyst indicate the possibility of magnetic recovery and reutilization without a significant loss of efficiency.

## 1. Introduction

Currently, water resource contamination by industry remains one of the main environmental issues [1,2]. The textile industry is one of the most polluting industries worldwide, not only considering the huge volumes of water consumed every day, but also the chemicals used in textile processing, such as dyes [3,4]. Effluents containing large amounts of dyes are discharged daily without completed treatment leading to a high negative impact on the ecosystem and also being a threat to public health considering their hazardous nature, persistence, and carcinogenic properties [5,6].

Photocatalysis has emerged as an efficient mechanism for the degradation of dyes [7,8,9]. In this process, a semiconductor absorbs energy (determined by the bandgap) by exciting electrons to the conduction band and forming electron/hole pairs. The latter can originate in reactive species (like the radicals ·O_2_^−^ or ·OH) that will react with dye molecules, converting them into inert products (e.g., water, carbon dioxide) [10,11]. Since about 43% of total solar radiation falls in the visible spectral region, the use of efficient photocatalysts absorbing visible light becomes a priority [12,13], also allowing for a decrease in energy consumption and associated costs [14,15].

Zinc/calcium mixed ferrites have appeared as interesting semiconductors for photocatalysis [16]: the incorporation of calcium improved both biocompatibility and magnetic properties compared to neat zinc ferrite [17,18] and pointed to a possible recovery and reuse of the magnetic nanoparticles. These nanoparticles present an estimated bandgap of 1.78 eV allowing for the use of visible light in effluent photoremediation [16]. However, ferrite nanoparticles present some limitations, namely the high electron/hole recombination rate giving low photodegradation rates [19]. Several investigations were developed using noble metals as coatings, to decrease the recombination rate, and to enhance the photocatalytic activity of the nanosystem [20,21], as the electron is transferred to the silver nanoparticles while the hole remains in the ferrite phase. Heterostructured materials such as AgCl on zirconium phosphate [22] and AgCl on ZnAl layered double hydroxide [23] were also reported to have improved the photocatalytic effect in dye photodegradation. This enhanced activity was shown to arise from the photoreduction of silver ions into silver nanoparticles, which enabled the population of the AgCl conduction band through visible photon absorption by the plasmonic silver nanoparticles. This process can also be considered a plasmonic-induced separation of electron-hole pairs with the hole on the metal and the electron on the wide bandgap AgCl semiconductor.

**Figure 1:**
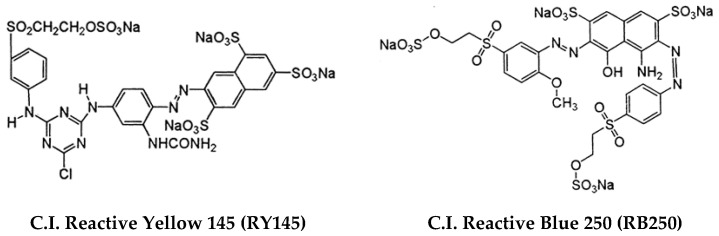
Structure of the industrial textile dyes used in photodegradation assays.

In this work, zinc/calcium mixed ferrites were prepared by the sol–gel method, as this method is relatively advantageous to co-precipitation concerning the nanoparticle’s size dispersion and crystallinity [21]. To enhance the photocatalytic activity, the mixed ferrites were coupled with silver nanoparticles [24,25] undergoing a photodeposition process with the aim of obtaining the improved characteristics of the photodegradation and reutilization of the photocatalyst. Rhodamine B was tested as model dye [26,27,28], while the industrial textile dyes C.I. Reactive Blue 250 and C.I. Reactive Yellow 145, resistant azo dyes (Figure 1), were chosen for a comparison with previous work [16], where a non-optimized nanosystem was unable to degrade these dyes. The results reported here were promising for the scale-up of the photocatalytic process and reutilization of the catalyst.

## 2. Materials and Methods

### 2.1. Preparation of Mixed Zinc/Calcium Ferrite Nanoparticles

Zinc/calcium ferrite nanoparticles, Zn_0.5_Ca_0.5_Fe_2_O_4_, were prepared by the sol–gel method adapting a protocol described by Samariya et al. [29]. A solution containing 20 mL of water, 807.9 mg of iron(III) nitrate nonahydrate, 73.5 mg of calcium chloride dihydrate, and 68.15 mg of zinc chloride was placed in a beaker under constant stirring. Then, 630.42 mg of citric acid as morphology controller and 70 μL of concentrated nitric acid were added to the solution. The solution was slowly heated at 90 °C of temperature until it formed a gel, and then further heated until it formed a loose powder. The as-prepared zinc/calcium ferrite nanoparticles were calcined for 30 min at 400 °C to improve their crystallinity. For comparison, zinc/calcium ferrite nanoparticles were also prepared by the co-precipitation method, as previously described [16].

### 2.2. Silver Coating by Photodeposition Method

The prepared zinc/calcium ferrite nanoparticles, either by the sol–gel or co-precipitation method, were coupled with silver by photodeposition adapting a method described by Liu et al. [30]. First, 30 mg of nanoparticles were dispersed in an aqueous solution under sonication for 30 min. Then, 1.5 mL of silver nitrate 1 M solution and 1 mL of methanol were added, followed by an irradiation for 12 h with a UV light lamp (200 W Xe-arc lamp, L.O.T.-Oriel GmbH & Co. KG, Darmstadt, Germany). After irradiation, the nanoparticles were washed with ultrapure water (Milli-Q grade) and dried for 12 h.

### 2.3. Structural Characterization

X-ray diffraction (XRD) measurements were performed in a conventional PAN’alytical X’Pert PRO diffractometer (Malvern Panalytical Ltd., Malvern, UK) operating with a CuK_α_ radiation in a Bragg Brentano configuration. Magnetization measurements were carried out in an MPMS3 SQUID magnetometer (Quantum Design Inc., San Diego, CA, USA). The hysteresis cycles (magnetization versus magnetic field) of the samples were measured in the convenient field range for each sample with a possible maximum ±50 kOe (±5 Tesla). The measurement method was by DC extraction or VSM oscillation at a frequency of 14 Hz. A specific magnetic field correction for the trapped flux in the superconducting coil was made achieving an accuracy of residual less than 2 Oe.

TEM images of nanoparticles were acquired using a transmission electron microscope JEOL 2100 (JEOL USA Inc., Peabody, MA, USA) operating at 200 kV. The solutions were sonicated in ethanol and dropped onto a TEM grid (copper 400 mesh with a carbon film). TEM images were processed using ImageJ software (National Institutes of Health (NIH), Bethesda, MD, USA), and the histograms were fitted to Gaussian distributions.

### 2.4. Photodegradation Assays

A home-built irradiation apparatus was used to evaluate the photocatalytic activity of the prepared samples in the degradation of aqueous solutions of the dyes Rhodamine B (40 mg/L), C.I. Reactive Yellow 145 (80 mg/L), and C.I. Reactive Blue 250 (80 mg/L). The setup incorporates a 200 W Xenon Arc Lamp (L.O.T.-Oriel GmbH & Co. KG, Darmstadt, Germany), a 400 nm long-pass filter (Thorlabs Inc., Newton, NJ, USA) to isolate the visible spectrum radiation, and a sample cuvette holder. Nanoparticles were dispersed, at a concentration of 2 mg/mL, in an aqueous solution of dye and allowed to equilibrate by magnetic stirring in the dark for 30 min. Absorption spectra of aliquots taken at given irradiation times and centrifuged to remove photocatalyst were measured in a Shimadzu UV-3600 Plus UV-Vis-NIR spectrophotometer (Shimadzu Corporation, Kyoto, Japan).

The kinetic constants of dye photodegradation can be estimated by applying a pseudo-first-order kinetic model (Equation (1)),

(1) $$\ln\left(C/C_{0}\right)=-kt$$

where *k* is the photodegradation rate constant (min^−1^), *C*_0_ is the initial concentration of the dye, and *C* is the concentration of the dye at different irradiation times, *t*.

## 3. Results and Discussion

### 3.1. Nanoparticles Characterization

It was reported that the coupling of photocatalytic nanoparticles with silver led to a decrease of a recombination of the generated electron/hole pairs with an enhanced formation of reactive species and an improved corresponding photocatalytic activity. This was already described for silver-doped zinc oxide [5], titanium dioxide [25,31], zinc ferrite [21], CdS nanoparticles [32], and ZnS nanoparticles [32]. In this work, we aimed at preparing optimized Zn_0.5_Ca_0.5_Fe_2_O_4_ nanoparticles with silver islands to potentiate their photocatalytic activity and allow the reuse of the photocatalyst. For that purpose, the sol–gel method was chosen for nanoparticle synthesis.

**Figure 2:**
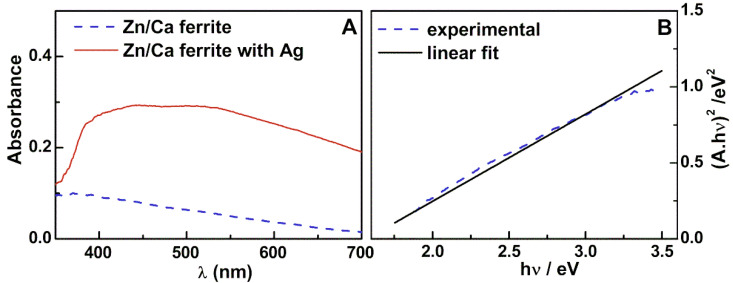
Absorption spectra of zinc/calcium ferrite obtained by the sol–gel method dispersed in water and with photodeposited silver (**A**) and Tauc plot for the ferrite nanoparticles (**B**).

The UV-visible absorption spectra of the prepared nanoparticles are shown in Figure 2A. Using a standard Tauc plot, a direct bandgap of 1.6 eV was determined for the zinc/calcium mixed ferrite nanoparticles (Figure 2B). This value is similar to the one obtained previously for the same type of ferrite prepared by the co-precipitation method [16]. The absorption spectrum of Zn/Ca mixed ferrite upon silver photodeposition (Figure 2A) clearly evidenced the presence of plasmonic bands from metallic silver, with a broad band starting below 400 nm and extending to the NIR spectral region. These features are compatible with the presence of either aggregated spherical silver nanoparticles [33] or silver nanodiscs [34].

**Figure 3:**
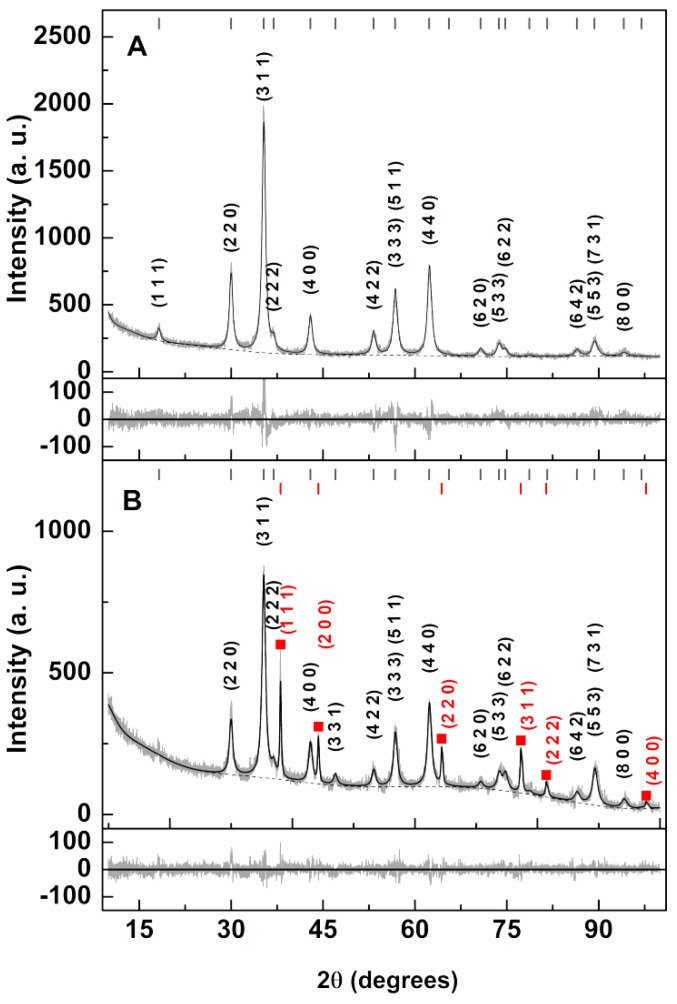
XRD diffractograms and corresponding Rietveld analysis of (**A**) Zn/Ca mixed ferrite and (**B**) the result of the silver photodeposition on its surface. The Miller indices of each peak are indicated (in black for Zn/Ca ferrite and in red for silver) and the silver phase is marked with red squares.

XRD results (Figure 3) confirm the crystallinity of the prepared nanoparticles. Profex software [35], which is based on BGMN [36], was used to conduct a Rietveld analysis of the experimental diffractograms. The crystal structure of the Zn/Ca mixed ferrite was defined through an adaptation of the zinc ferrite CIF file nr. 2,360,015 (space group Fd-3m:1) considering a stoichiometric distribution of cations across the tetrahedral and octahedral sites and assuming an inversion degree of 1 [17]. The silver phase was accounted by the use of CIF file nr. 9,008,459. In Table 1, the main results obtained by the Rietveld analysis are shown.

Reasonable fits with R_P_ values of 7.91 and 5.89, respectively, were obtained from the samples with and without silver. The mixed ferrite lattice parameter was similar to the one obtained from similar particles prepared by the co-precipitation method [16]. The implementation of a size broadening effect in BGMN allowed an estimation of 12.4 nm for the ferrite phase and 42.4 nm for the silver nanostructures.

The magnetic properties of calcium-substituted zinc ferrite nanoparticles resulted from the cation distribution along their spinel structure. The magnetic moment of Fe^3+^ cations is 5 µ_B_ while Zn^2+^ and Ca^2+^ are non-magnetic. Additionally, Ca^2+^ is a large cation with an ionic radius of 0.99 Å, having a strong influence on the distributions of magnetic ions in interstitial sites. The spinel structure of calcium-substituted zinc ferrites could be written as ($Ca_{x}^{2+}Zn_{y}^{2+}Fe_{1-x-y}^{3+})\left[Ca_{x}^{2+}Zn_{y}^{2+}Fe_{1+x+y}^{3+}\right]O_{4}^{2-}$, where the round and square brackets represent tetrahedral (A) and octahedral [B] sites, respectively; *x* and *y* denote the inversion degree as the fraction of the (A) sites are occupied by Fe^3+^. Both divalent cations, Zn^2+^ and Ca^2+^, had a tetrahedral (A) site preference [17,37], anticipating a reduction of exchange interactions between the cation’s occupancy in both tetrahedral and octahedral sites. Thus, the net magnetization was expected to decrease with a possible migration of the divalent cations to the octahedral site.

**Figure 4:**
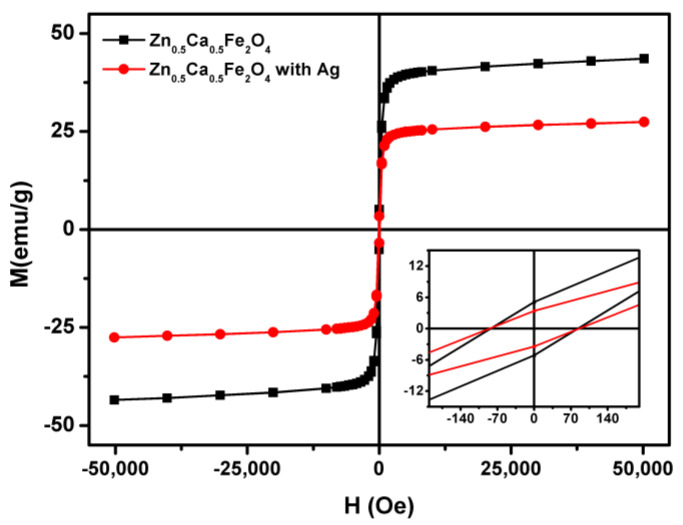
Hysteresis loop of Zn_0.5_Ca_0.5_Fe_2_O_4_ and Zn_0.5_Ca_0.5_Fe_2_O_4_ with Ag nanoparticles at room temperature. Inset: Enlargement in the low field region of the loop.

Figure 4 displays the magnetic hysteresis loops of the synthesized nanoparticles at room temperature. These measurements gave information about the saturation magnetization (M_s_), the degree at which the sample remains magnetized after the applied field was removed (the remanent magnetization, M_r_), and how easily the sample magnetization could be reversed, the so-called coercive field (C). The magnetic properties are summarized in Table 2.

Using the sol–gel preparation technique, the maximum magnetization of zinc/calcium mixed ferrite rose more than two times compared with the similar, previously observed nanoparticles prepared by the co-precipitation method (M_s_ = 20.45 emu/g [16]), despite their significantly higher coercivity. This evidenced a notable improvement in the magnetic properties of the ferrites, which was important for the purpose of the magnetic recovering (and reuse) of the photocatalyst. Regarding the silver-doped nanoparticles, as expected, the saturation magnetization is lower, due to the presence of a non-magnetic silver coating. Jasso-Terán et al. [17] reported Zn_0.5_Ca_0.5_Fe_2_O_4_ nanoparticles of about 14 nm and a saturation magnetization of 31.31 emu/g, also prepared by the sol–gel methodology. Here, for a similar size, a higher maximum magnetization was reached.

Both Zn_0.5_Ca_0.5_Fe_2_O_4_ and Zn_0.5_Ca_0.5_Fe_2_O_4_/Ag nanoparticles showed to be in the limit for a superparamagnetic behavior, with magnetic squareness values, M_r_/M_s_, of about 0.1 (Table 2). If below 0.1, this ratio indicated that more than 90% of the magnetization was lost upon the removal of the applied magnetic field and the nanoparticles were superparamagnetic. The low field region enlargement (inset of Figure 4) revealed a slightly opened curve with coercivity values of 82.66 Oe and 85.43 Oe for Zn_0.5_Ca_0.5_Fe_2_O_4_ and Zn_0.5_Ca_0.5_Fe_2_O_4_/Ag nanoparticles, respectively.

**Figure 5:**
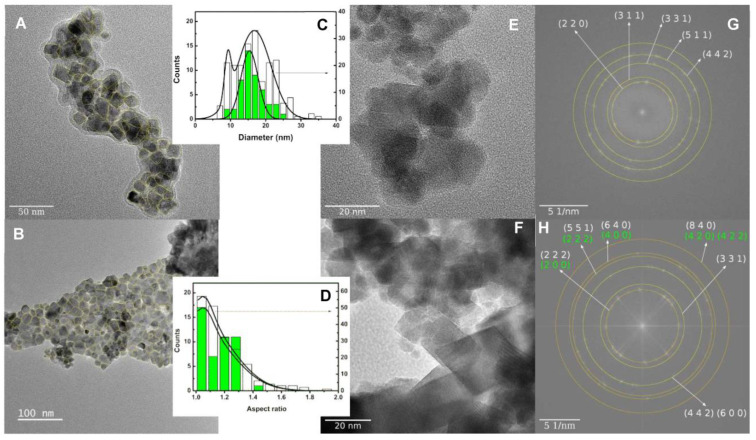
TEM images of a plain Zn/Ca mixed ferrite (**A,E**) and coupled with silver (**B,F**). Size (**C**) and aspect ratio (**D**) histograms resulting from outlined particles where filled bars are for plain mixed ferrite. Panels (**G**) and (**H**) show the fast Fourier transform (FFT) of, respectively, images E and F, and include the identified electron diffraction rings from their Miller indices for Zn/Ca ferrite spinel (white) and silver fcc (yellow) crystal structures.

Transmission electron microscopy (TEM) imaging results of the prepared nanoparticles are presented in Figure 5.

Zn/Ca mixed ferrites (Figure 5A,E) exhibited a cubic-like structure and the silver photodeposition process seemed to introduce smaller round particles (Figure 5B,F). Size estimation was achieved by manually outlining the particles and considering the diameter of a circle with an equivalent area. The corresponding histogram (Figure 5C) showed that the size of the mixed ferrite nanoparticles is 15 ± 2 nm, which got enlarged upon the silver photodeposition process into 17 ± 5 nm. As the XRD results showed no significant size variation of the ferrite phase upon silver photodeposition, the increase seen here either corresponded to an enhanced nanoparticle aggregation or to an increase in size by silver coupling to the mixed ferrite nanoparticles. Nevertheless, the estimations of the size of the ferrite phase from TEM and XRD are compatible. There was also a noticeable appearance of a smaller size population (9.4 ± 0.9 nm) in the nanoparticles obtained by the silver photodeposition process. This was tentatively assigned to silver nanoparticles coupled with the ferrite surface, as observed in a previous study using a different silver deposition process [16]. Yet, the XRD size prediction of the silver phase was much higher (42.4 nm). This discrepancy could be due to the influence of a specific topology of the nanoparticles (silver on ferrite surface), as it was already observed in other metal coupled nanostructures, namely a gold diffraction peak width from a 2 nm shell equal to the one from a 10 nm magnetite core [38]. Using ImageJ software, it was possible to fit each outlined particle in a rectangle. Taking the ratio between the longer and smaller side, it resulted in an aspect ratio parameter that is represented in Figure 5D taking values mainly up to 1.4.

Further confirmation of the structure of imaged nanoparticles in Figure 5E,F could be obtained through fast Fourier transform (FFT) showing spots that resulted from periodic variations of the TEM images. These were analyzed by measuring the radius in the Fourier space of the circles that overlap in diffraction spots. The inverse of such a radius was the distance of adjacent lattice planes, d_hkl_, where (h, k, and l) are Miller indices. For Figure 5G, using the spinel crystal structure and the lattice parameter obtained from the XRD measurements, it was possible to obtain a calibration factor so that the observed rings were compatible with the diffraction from the mixed Zn/Ca ferrite. Using the same calibration factor in the analysis of Figure 5H, some of the rings were also ascribable to the fcc (face centered cubic) structure of silver with the lattice parameters obtained from the XRD measurements. This analysis gave an indication of the presence of silver nanoparticles in Figure 5F probably corresponding to the circular-like areas.

### 3.2. Photodegradation Assays

**Figure 6:**
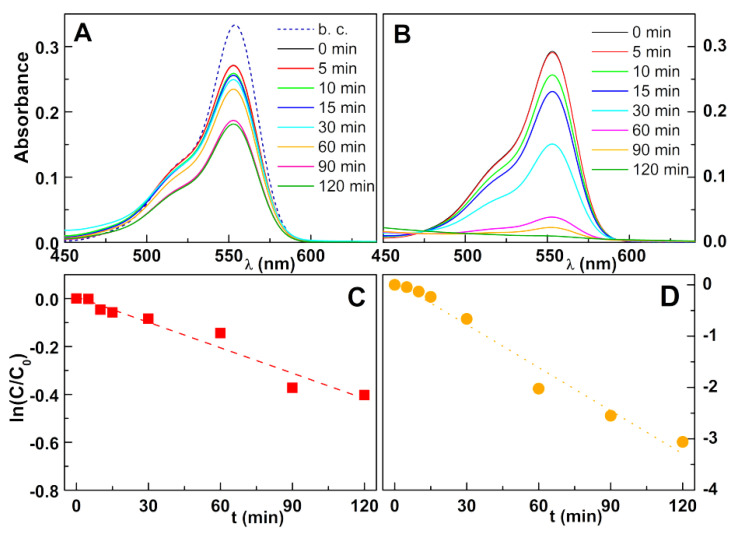
(**A**,**B**): Photodegradation assays of solutions of the dye Rhodamine B (40 mg/L) by zinc/calcium ferrite nanoparticles covered with silver by photodeposition. b. c.: Dye absorption spectrum before adding the catalyst; (**A**): Ferrites prepared by co-precipitation; (**B**): Ferrites prepared by sol–gel. (**C**,**D**): Plot of the pseudo-first-order kinetics for degradation of Rhodamine B; (**C**): Ferrites prepared by co-precipitation; (**D**): Ferrites prepared by sol–gel.

Rhodamine B is widely employed as a model dye in photodegradation studies and was also used here for comparison purposes. To clarify the effect of the preparation method of mixed zinc/calcium ferrites, ferrite nanoparticles prepared by either sol–gel or co-precipitation were compared. Clearly, the sol–gel preparation method was advantageous for the degradation of Rhodamine B by silver-photodeposited coated nanoparticles (Figure 6) with a total degradation after 120 min (Figure 6B), while for nanoparticles prepared by co-precipitation, the degradation attained only 35% (Figure 6A). First-order kinetics was followed in both cases (Figure 6 C,D), with rate constants in Table 3. The results previously obtained for nanoparticles prepared by co-precipitation and silver deposited by a reflux procedure [16] were also shown for direct comparison. In the case of Rhodamine B, the higher rate constant was half of the previously observed in nanoparticles with silver coating obtained by reflux, probably due to a higher content of silver on the latter, and to a higher particle load (3 mg/mL).

**Figure 7:**
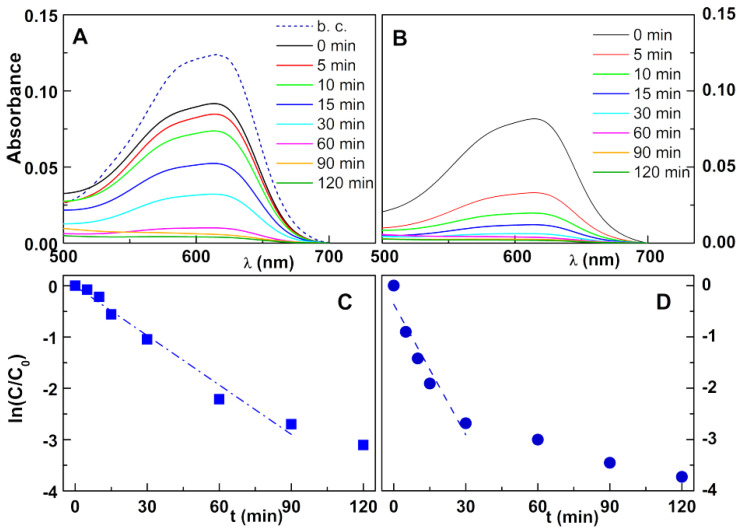
(**A**,**B**) Photodegradation assays of solutions of the textile dye Reactive Blue 250 (80 mg/L) by zinc/calcium ferrite nanoparticles covered with silver by photodeposition; b. c.: Dye absorption spectrum before adding the catalyst; (**A**) Ferrites prepared by co-precipitation; (**B**) Ferrites prepared by sol–gel. (**C**,**D**) Plot of the pseudo-first-order kinetics for degradation of Reactive Blue 250; (**C**) Ferrites prepared by co-precipitation; (**D**) Ferrites prepared by sol–gel.

The developed nanoparticles were also assayed in the degradation of the textile industry dyes Reactive Blue 250 and Reactive Yellow 145. For Reactive Blue 250 (Figure 7), a total degradation was obtained with zinc/calcium ferrites obtained by sol–gel and photodeposition of silver (after 9 min) with a strong decrease in dye content in the first 5 min (Figure 7B). In this latter case, a first-order kinetics was obtained only until 30 min, as almost all dye was in fact degraded in this time interval.

**Figure 8:**
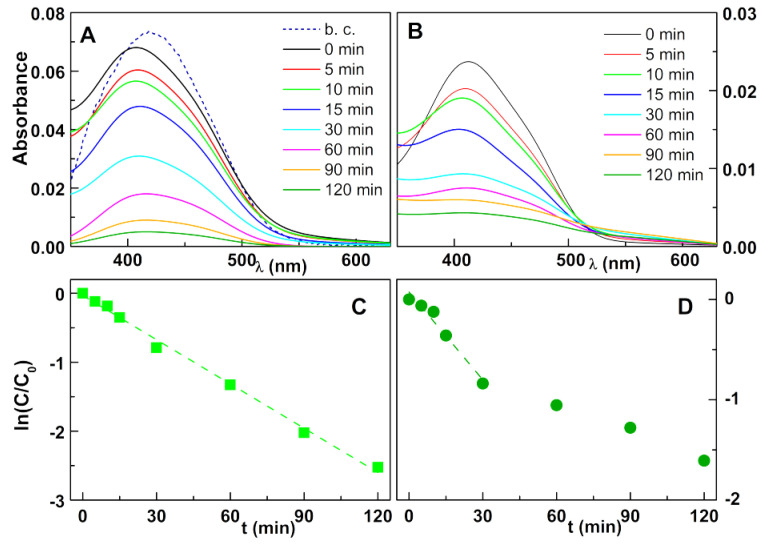
(**A**,**B**) Photodegradation assays of solutions of the textile dye Reactive Yellow 145 (80 mg/L) by zinc/calcium ferrite nanoparticles covered with silver by photodeposition; b. c.: Dye absorption spectrum before adding the catalyst; (**A**) Ferrites prepared by co-precipitation; (**B**) Ferrites prepared by sol–gel. (**C**,**D**) Plot of the pseudo-first-order kinetics for degradation of Reactive Yellow 145; (**C**) Ferrites prepared by co-precipitation; (**D**) Ferrites prepared by sol–gel.

Regarding the Reactive Yellow dye, the degradation is less efficient. For the ferrites prepared by co-precipitation, the degradation was gradual until 120 min (Figure 8A,C). For the sol–gel nanoparticles (Figure 8B,D), a fast degradation was observed until 30 min, with a decrease in rate thereafter.

The obtained rate constant for Reactive Blue 250 using Zn_0.5_Ca_0.5_Fe_2_O_4_ (sol–gel)/Ag (photodeposition) nanoparticles was similar to the reported value (*k =* 0.0885 min^−1^) for Reactive Blue 4 using pure anatase nano-TiO_2_ under UV light [39]. Regarding RY145, the degradation rates were much lower but were higher than the value reported for this dye using a Ni_3_O_4_-Co_3_O_4_/Al_2_O_3_ catalyst when pseudo-first-order kinetics was followed (*k =* 0.01623 min^−1^) [40]. The different degradation rates of the two reactive azo dyes must be related to chemical structure variations, as already observed in the photodegradation of azo dyes using ZnO nanoparticles [41]. This different reactivity also manifests itself as distinct adsorption effects on absorption spectra, when comparing particles obtained from co-precipitation or sol–gel methods, where similar variations were observed for the blue dye (Figure 7A,B) while an enhanced absorption decrease was exhibited in sol–gel nanoparticles for the yellow dye (Figure 8A,B). The main structural difference was the existence of an s-triazine ring in the yellow dye, which was probably responsible for the distinct behavior. As referred in the Introduction section, the degradation mechanism was mainly determined by the action of reactive species like the ·O_2_^−^ or ·OH radicals, which were formed during the irradiation by the catalytic effect of the nanoparticles studied. The ·OH could result from the reaction of the holes, $h_{VB}^{+}$, with adsorbed OH^−^ ions or water molecules. However, this was not possible for the used photocatalyst, as the valence band position for ZnFe_2_O_4_ and CaFe_2_O_4_ was 0.38 eV [42] and 0.42 eV [43], respectively, whereas the reduction potentials for ·OH/OH^−^ and ·OH/H_2_O were 1.99 V [42] and 2.31 V [44], respectively. The superoxide anion (·O_2_^−^) originated from the transfer of conduction band electrons ($e_{CB}^{-}$) by dissolved oxygen. As the reduction potential for O_2_/·O_2_^−^ is −0.18 V [44] and the conduction band position for zinc/calcium ferrite was calculated to be between −1.22 eV and −1.18 eV, the production of superoxide was very favorable. In the previous discussion, all the values of energy and reduction potentials were relative to NHE (normal hydrogen electrode). This superoxide radical could originate ·OH through the formation of H_2_O_2_ using further conduction band electrons [42]. Although the valence band position was not very positive, direct oxidation of adsorbed molecules was also possible [42]. Thus, the main reactive species were the superoxide, the ·OH (indirectly produced via superoxide), and $h_{VB}^{+}$. This was proved using suitable scavengers for both CaFe_2_O_4_ [43] and ZnFe_2_O_4_ [45]. The effect of silver decorated nanoparticles was a decrease of the possibility of electron-hole recombination by acting as a sink for the ferrite conduction band electrons. Additionally, it could also enhance the transfer of electrons to oxygen. Then, the following photodegradation mechanism was expected:

$${\mathrm{Zn}}_{0.5}{\mathrm{Ca}}_{0.5}{\mathrm{Fe}}_{2}O_{4}\;+\;h\nu \;\to \;{\mathrm{Zn}}_{0.5}{\mathrm{Ca}}_{0.5}{\mathrm{Fe}}_{2}O_{4}(e_{CB}^{-})+\;{\mathrm{Zn}}_{0.5}{\mathrm{Ca}}_{0.5}{\mathrm{Fe}}_{2}O_{4}(h_{VB}^{+})$$

$${\mathrm{Zn}}_{0.5}{\mathrm{Ca}}_{0.5}{\mathrm{Fe}}_{2}O_{4}(e_{CB}^{-})\;+\;{\mathrm{Zn}}_{0.5}{\mathrm{Ca}}_{0.5}{\mathrm{Fe}}_{2}O_{4}(h_{VB}^{+})\to {\mathrm{Zn}}_{0.5}{\mathrm{Ca}}_{0.5}{\mathrm{Fe}}_{2}O_{4}$$

$${\mathrm{Zn}}_{0.5}{\mathrm{Ca}}_{0.5}{\mathrm{Fe}}_{2}O_{4}(e_{CB}^{-})\;+\;O_{2}\;\to \;{\mathrm{Zn}}_{0.5}{\mathrm{Ca}}_{0.5}{\mathrm{Fe}}_{2}O_{4}+\cdot {O_{2}}^{-}$$

$${\mathrm{Zn}}_{0.5}{\mathrm{Ca}}_{0.5}{\mathrm{Fe}}_{2}O_{4}(h_{VB}^{+})\;+\;\mathrm{Dye}\;\to \;{\mathrm{Zn}}_{0.5}{\mathrm{Ca}}_{0.5}{\mathrm{Fe}}_{2}O_{4}\;+\;(\mathrm{photodegradation}\;\mathrm{products})$$

$${\mathrm{Zn}}_{0.5}{\mathrm{Ca}}_{0.5}{\mathrm{Fe}}_{2}O_{4}(e_{CB}^{-})+\cdot {O_{2}}^{-}+2H^{+}\to {\mathrm{Zn}}_{0.5}{\mathrm{Ca}}_{0.5}{\mathrm{Fe}}_{2}O_{4}+H_{2}O_{2}$$

$${\mathrm{Zn}}_{0.5}{\mathrm{Ca}}_{0.5}{\mathrm{Fe}}_{2}O_{4}(e_{CB}^{-})\;+\;H_{2}O_{2}\;\to \;{\mathrm{Zn}}_{0.5}{\mathrm{Ca}}_{0.5}{\mathrm{Fe}}_{2}O_{4}+\cdot OH+OH^{-}$$

Dye + ·OH → (photodegradation products)

$$\mathrm{Dye}\;+\;\cdot {O_{2}}^{-}\;\to \;(\mathrm{photodegradation}\;\mathrm{products})$$

Overall, the results pointed to a significant rise in photodegradation efficacy using zinc/calcium ferrites prepared by sol–gel and coated with photodeposited silver in the case of blue and yellow industrial dyes, relative to previous work [16] (where these two dyes were hardly degraded) and the systems that used UV-light reported in the literature.

**Figure 9:**
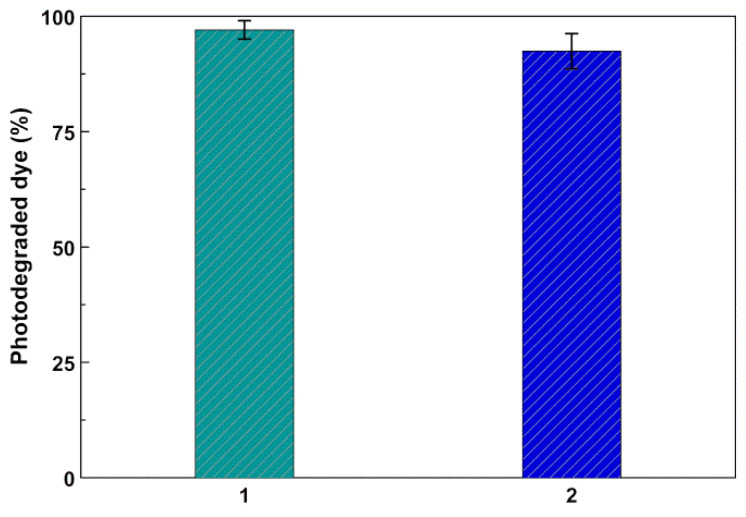
Percentage of degraded dye for Reactive Blue 250 in two cycles (1 and 2) of use of the photocatalyst. Error bars from three assays.

The possibility of reutilization of the photocatalyst was an important feature of the magnetic-nanoparticles-based materials for effluent remediation. This way, a two-cycle assay was performed using the Reactive Blue 250 dye and the more active photocatalyst Zn_0.5_Ca_0.5_Fe_2_O_4_ (sol–gel)/Ag (photodeposition). After the first cycle of degradation for 120 min, the catalyst was magnetically removed and washed, and no loss of mass was detected. The photodegradation assay was then repeated (Figure 9). Only a slight loss of catalyst activity could be observed with the percentage of the degraded dye in the second cycle being higher than 90%. This result pointed to promising reuse of this photocatalyst in industrial effluent remediation by taking advantage of the magnetic recovery.

## 4. Conclusions

In this work, zinc/calcium mixed ferrite nanoparticles were synthesized by a sol–gel method and coupled to silver clusters by photodeposition. Upon silver photodeposition, it was found that the sol–gel method originated ferrite nanoparticles with superior photocatalytic properties towards Rhodamine B photodegradation. The developed nanoparticles were also successfully employed in the photodegradation of industrial textile azo dyes showing, for all, full degradation using visible light, whereas, in a previous study [16] only the red azo dye was completely photodegraded. The magnetic properties of the nanoparticles were exploited for easy isolation of the photocatalyst, and no loss of photocatalytic response upon reuse was demonstrated.

## Figures and Tables

**Table 1:** Selected parameters from the Rietveld analysis using BGMN.

Sample	O_x,y,z_ (*)	*i* (*)	Phase Size (nm) Lattice Constant (nm) Zn/Ca Ferrite|Ag	Ag (wt%)	R_P_	χ^2^
Zn/Ca ferrite	0.3834	1 (^+^)	12.3 | ---- 0.8418 | ----	-----	5.89	1.16
Zn/Ca ferrite with silver photodeposition	0.3505	1 (^+^)	12.4 | 42.4 0.8418(+) | 0.409	4.31	7.91	1.87

(*) Value of O_x,y,z_ in CIF file 2,300,615 is 0.2535. (^+^) fixed value.

**Table 2:** Coercive field (C), saturation magnetization (M_s_), remanent magnetization (M_r_), and ratio M_r_/M_s_ for zinc/calcium ferrites at room temperature.

Nanoparticles	M_s_ (emu/g)	M_r_ (emu/g)	C (Oe)	M_r_/M_s_
Zn_0.5_Ca_0.5_Fe_2_O_4_ (sol–gel)	44.22	5.03	82.66	0.11
Zn_0.5_Ca_0.5_Fe_2_O_4_ (sol–gel)/Ag (photodeposition)	27.97	3.31	85.43	0.12

**Table 3:** Rate of degradation of the dyes in a pseudo-first-order kinetics.

	*k* (min^−1^)		
	Rhodamine B	RB250	RY145
Zn_0.5_Ca_0.5_Fe_2_O_4_ (co-precipitation)/Ag (photodeposition)	0.0035	0.0321	0.0214
Zn_0.5_Ca_0.5_Fe_2_O_4_ (sol–gel)/Ag (photodeposition)	0.0310	0.0847	0.0292
Zn_0.5_Ca_0.5_Fe_2_O_4_ (co-precipitation)/Ag (reflux) [16]	0.0614	0.0104	0.0058

## Data Availability

Not applicable.
